# Psycho-education for substance use and antisocial personality disorder: a randomized trial

**DOI:** 10.1186/s12888-015-0661-0

**Published:** 2015-11-14

**Authors:** Birgitte Thylstrup, Sidsel Schrøder, Morten Hesse

**Affiliations:** Centre for Alcohol and Drug Research, Aarhus University, Artillerivej 90, 2nd, 2300 Copenhagen S, Denmark

## Abstract

**Background:**

Antisocial personality disorder often co-exists with drug and alcohol use disorders.

**Methods:**

This trial examined the effectiveness of offering psycho-education for antisocial personality disorder in community substance use disorder treatment centers in Denmark. A total of 176 patients were randomly allocated to treatment as usual (TAU, *n* = 80) or TAU plus a psycho-educative program, Impulsive Lifestyle Counselling (ILC, *n* = 96) delivered by site clinicians (*n* = 39). Using follow-up interviews 3 and 9 months after randomization, we examined changes in drug and alcohol use (Addiction Severity Index Composite Scores), percent days abstinent (PDA) within last month, and aggression as measured with the Buss-Perry Aggression Questionnaire-Short Form and the Self-Report of Aggression and Social Behavior Measure.

**Results:**

Overall engagement in psychological interventions was modest: 71 (76 %) of participants randomized to psycho-education attended at least one counselling session, and 21 (23 %) attended all six sessions. The Median number of sessions was 2. All patients reduced drug and alcohol problems at 9 months with small within-group effect sizes. Intention-to-treat analyses indicated significant differences between ILC and TAU in mean drugs composite score (*p* = .018) and in PDA (*p* = .041) at 3 months. Aggression declined in both groups, but no differences between ILC and TAU were observed in terms of alcohol problems or aggression at any follow-up.

**Conclusions:**

Moderate short-term improvements in substance use were associated with randomization to Impulsive Lifestyle Counselling. The findings support the usefulness of providing psycho-education to outpatients with antisocial personality disorder.

**Trial registration:**

ISRCTN registry, ISRCTN67266318, 17/7/2012

**Electronic supplementary material:**

The online version of this article (doi:10.1186/s12888-015-0661-0) contains supplementary material, which is available to authorized users.

## Background

Antisocial personality disorder (ASPD) is a serious disturbance that imposes a major burden on individuals and society [27] and for which there is no effective treatment [25]. Affected individuals exhibit persistent antisocial behavior and pervasive antisocial character traits, such as irritability, manipulativeness, and lack of remorse [2]. The disorder affects between 1.0 and 3.6 % of the general population (e.g. [11, 13]), and a substantially greater percentage of patients with substance use disorders [13]. In terms of developmental psychopathology, there is evidence that ASPD is a lifespan disorder which originates in childhood [20], and accordingly, the presence of conduct disorder is a prerequisite to the adult diagnosis [2].

### Treatments for ASPD

Early intervention for children with antisocial behavior may prevent the development of ASPD and improve academic performance [46]. As for treatment for adults, the treatment options that have been tested have been designed to treat comorbid substance use disorders (SUDs) or have targeted a specific behavior such as social problem-solving skills. Overall, evidence for the efficacy of psychosocial interventions is weak [25]. In terms of mental health service use, individuals with ASPD have a high risk of needing emergency psychiatric services and inpatient hospitalization [15]. Although they are not likely to seek treatment for their behavioral problems, many patients with ASPD do seek treatment for drug and alcohol problems [24, 50]. Among individuals with SUD, ASPD is a common comorbidity across classes of substances [26] and is associated with poor prognosis [14, 33], even years or decades after a diagnosis is made ([21–23].

The only published controlled trial of which we are aware which has tested an intervention directed at any type of outpatients with ASPD, found a non-significant trend favoring cognitive behavior therapy over treatment as usual in terms of alcohol problems, but not in other outcomes, such as self-reported aggression and social functioning [16]. However, a potentially important observation from this controlled trial was that a substantial proportion of the participants were willing to engage in treatment and that it is possible to offer treatment to individuals with ASPD.

### Psycho-education

One way to address a personality disorder (PD) is through psychoeducation. If done sensitively, providing psychoeducation may help raise the patient’s awareness of his behavioral difficulties and how they impact himself and others [6]. This awareness may in turn help the patient in making informed decisions about seeking and receiving help for problems.

Paradoxically, many clinicians report not providing psychoeducation to patients with PDs, although they perceive it to be an important aspect of treatment [44]. Whether or not this applies to other treatment services and to ASPD is not known, but we are aware of no published manuals or studies on how to provide psychoeducation for patients with ASPD.

At present, there is no evidence on psychoeducation for ASPD, and little on psycho-education with patients with PD in general. A study of patients with borderline personality disorder (BPD) found that psychoeducation had an effect on impulsivity and chaotic interpersonal relationships, but not on global functioning [56].

While BPD and ASPD are two distinct disorders, they share central features such as impulsivity and high levels of anger; therefore it is possible that these findings could apply to the psychoeducation of patients with ASPD as well. If patients with ASPD gain a better understanding of their own personalities, they may identify dysfunctional beliefs and behaviors that emerge in various situations, such as the belief that it is necessary to dominate and control others [8, 38], and make informed decisions about how to change their behavior. Another study of psychoeducation with patients with PDs was conducted in two settings - community and forensic. The study did not have a control group, but after the course of psychoeducation, patients reported that therapeutic alliance improved [6].

Given that ASPD is highly prevalent among people with SUD, one context for reaching patients with ASPD is at substance use treatment services. Some studies have indicated that treatments that integrate PD as 2an important component in SUD treatment may reduce substance abuse among patients with comorbid SUD and PD when compared to substance abuse treatment alone, although the integration appears to have little effect on symptoms and functioning [3–5, 40]. Providing treatment to people with comorbid ASPD and SUD may be a challenge; in general, there is a risk of low attendance to psychotherapeutic treatments and counselling at community-based treatment of substance use disorders [17, 43], and this risk is exacerbated in patients with ASPD (e.g. [48]). In conclusion, there is a need for further development of clinical strategies that can address the types of problems associated with ASPD in settings where ASPD is common, such as substance abuse treatment centers.

### Aims

The purpose of this study was to test the efficacy of a brief psycho-educational intervention, the Impulsive Lifestyle Counselling program (ILC), as a supplement to standard substance abuse treatment in a regular outpatient context. The trial was pragmatic in the sense that the aim was to test the usefulness of implementing a brief intervention with limited demands on costs and clinician qualifications in a standard setting, community substance abuse treatment [57], in a way that is similar to a number of recent studies [17, 54].

## Methods

### Study design

The study was a Phase I pragmatic randomized controlled trial with single blind assessments, and was carried out at community-based substance abuse treatment clinics in 13 municipalities in Denmark between January 2012 and July 2014. Inclusion criteria were: between 18 and 65 years old; met criteria for ASPD using the Mini International Neuropsychiatric Interview [47], able to provide written informed consent, and seeking treatment or already in treatment for a substance use disorder. Exclusion criteria were: plans that would interfere with participation in the psychoeducation in the next three months, such as plans to move away from the uptake area or waiting to serve a prison sentence, plans to enter residential rehabilitation or hospitalization, and waiting to serve a prison sentence. Additionally, patients were excluded if they were participating in group counselling or therapy with another patient participating in the trial, were known to suffer from an acute psychosis or severe brain damage, or did not speak Danish.

### Ethics

The present project was reviewed by the regional ethics committee of the Capital Region of Denmark and deemed exempt from a formal evaluation (J#H-3-2012-FSP45). This study was done in accordance with the declaration of Helsinki 2004, which states that it is the duty of the researcher to protect the life, health, dignity, integrity, right to self-determination, privacy and confidentiality of personal information of research subjects (WMA, 2013). All patients signed separate consent forms to participate in the study and to be followed up. The Danish Data Protection Agency evaluated data security for the project and approved the procedures for data handling and storage. The trial was registered in the ISRCTN register (#ISRCTN67266318).

### Recruitment and randomization

Study participants were identified by clinicians at the participating sites from new and existing patients receiving outpatient treatment for a drug or alcohol problem. After agreeing to be contacted, relevant participants were invited by a trained clinician at the site to take part in an interview to assess the diagnosis of ASPD and the other inclusion criteria. Those participants who met the inclusion criteria were told that their responses indicated ASPD, and the counsellor would then review their responses to the MINI module and ask if they felt that the behavior described in the response constituted a problem to them, and if they were willing to speak about it with to a counsellor. Those who agreed to speak with a counsellor and provided written informed consent to take part in the study, subsequently completed the baseline assessment and were randomly allocated to either one of two active treatment groups: treatment as usual (TAU) or the Impulsive Lifestyle Counselling (ILC). Patients who consented to participate in the study were also asked to provide information for follow-up, including telephone numbers, home address, the addresses and telephone numbers of family members or others who could help locate the patient for the follow-up. Patients were also asked to specify which of a number of alternative contact sources other than the treatment clinic (prison services, social services, hospitals, homeless services) that they would consent to being used to locate them .

Randomization was stratified by clinic. The randomization schedules were generated by the trial coordinator and kept secure and confidential at the study coordinating center in Copenhagen. The randomization schedule was constructed using the method of randomized permuted blocks of randomly varying size with a ratio of 1:1 (4 or 6 per block).

The trial coordinator informed the referring clinician of the result of randomization immediately after being notified that the patient had been assessed and was found to be eligible for study participation. After this, the clinician informed the patient of the result. In the cases in which patients were randomized to the ILC treatment, the clinician then contacted one of the ILC counsellors at the uptake unit with the participants’ details so that the sessions could be initiated as quickly as possible.

Because the randomization had to take place immediately after the assessment interview, the trial coordinator was unable to check whether the baseline assessment was complete before randomizing, and patients with incomplete data at baseline had to be excluded after randomization.

### Treatment conditions

#### TAU

All participants received whichever form of treatment they would have received at the participating treatment service if the trial had not taken place. Treatment always included: access to opioid substitution treatment (either methadone, buprenorphine or a combination of methadone and injectable diacetylmorphine) for patients who needed it, and psychosocial support in the form of casework, counselling, or referral to residential rehabilitation. At some clinics, a liaison psychiatrist would see the patients on-site, and at other clinics patients would be referred to an off-site psychiatrist for diagnosis and treatment of other psychiatric conditions, such as attention-deficit/hyperactivity disorder, anxiety or depression.

#### ILC

In addition to all of the services available to patients who received TAU, patients randomized to ILC were offered up to six ILC sessions by a specially trained counsellor. The ILC program is a highly structured, manual guided psychoeducational intervention for people with ASPD [49]. Each session covers a specific topic and includes questions that the patient must be asked. The form and content of the sessions were adapted from the manual for the Lifestyle Issues program [53]. In line with the Lifestyle Issues program, the key is to support the patient in awareness raising, in recognizing the opportunity to change lifestyle and in taking responsibility for addressing behavioral problems. Similar to the approach by Banerjee and colleagues, the psychoeducational intervention is intended to function as a an educative and collaborative exercise that can improve further treatment engagement [6].

Each session covers a specific topic and includes questions that the patient must be asked, and pre-printed handouts and worksheets are given to the patient. The initial session focuses on the purpose of the ILC program and on identifying thoughts and behavior related to ASPD. The second session is based on an adapted version of the Antecedents-Beliefs-Consequences model from Rational-Emotive Behavior Therapy (Ellis & Dryden, 1997), linking the patients’ impulsive behaviors to the immediate consequences. Session 3 deals with impulsive and destructive behavior and how it may be related to specific value systems and beliefs associated with ASPD. Session 4 presents the concept of values and discusses which values may support or prevent the patient in change of lifestyle, and session 5 focus on the patient’s social networks and how certain people or groups may support or challenge lifestyle changes. The last session is a booster session in which the patient is invited to talk about the topics that he or she finds most relevant for future efforts to change behavior.

Like the Lifestyles Issues program, the ILC program is designed so that no prior training or special facilities of any sort are necessary. However, prior to delivering the intervention, all of the counsellors participated in two-day workshops to practice the strategies described in the manual and discuss issues related to treating people with ASPD in general. All counsellors were required to keep written records and make audio-recordings of the sessions.

Counsellors in the ILC group did not receive any special supervision beyond the supervision that was already available to staff in their respective clinics, but they did have the opportunity to call the study organizers with specific questions concerning the intervention.

### Follow-up procedures

For the two follow-up waves, patients were initially contacted through the phone number they had provided. If it was not possible to establish contact with the person, the next attempt was to contact the patient through the clinic at which they had been screened for the study. If a patient still could not be reached, we asked his or her case manager at the clinic if there was a time when the patient was expected to be at the clinic (e.g., times when the patient would pick up medications). Patients who could still not be reached were contacted through the telephone numbers and addresses they had provided, and finally through other available sources that the patient had given consent to at the study intake. In a few cases, the patients were finally located through the Central Personal Register. Once a patient had been located, and if it was possible to speak with the patient directly, a place and time for an interview was scheduled. If the patient did not show up for a face-to-face interview, a new time would be scheduled, and only after several failed attempts was a telephone interview suggested. If the patients stated that they were not willing to be interviewed, they would be asked if they would agree to be contacted at a later point, and if they refused, they were not contacted again.

## Measures

The Mini International Neuropsychiatric Interview [MINI] ASPD module was used to assess ASPD [31, 47]. The MINI is a fully structured, brief and valid diagnostic interview that was designed to assess DSM-IV and ICD-10 diagnoses [31, 47], which can be conducted by a lay person and is well accepted by patients [41]. The ASPD module consists of six questions concerning conduct disorder and six questions about adult antisocial behavior. Previous research indicates that the MINI module for ASPD identifies prison inmates with more serious mental health problems, more substance abuse problems, a more serious and chronic history of offending behavior compared with other inmates [9, 32, 35], and is associated with illicit drug use in the general population [37]. For the present study, we used the official Danish translation of the MINI 5.0.0 by P. Besh, G. Bech-Andersen, and T. Schütze. Also, after each adult antisocial item on the MINI schedule, staff members asked about whether the behavior had occurred in the past year, in order to confirm that the behavior was ongoing. The sample internal consistency of the lifetime adult antisocial behavior items was Cronbach’s α = 0.74 at baseline and α = 0.65 for the conduct disorder criteria.

Additional demographic data were collected on a separate sheet, including education, employment history, history of homelessness, residential treatment for substance use disorder, incarceration, and psychiatric hospitalizations.

Current substance use severity was measured using the alcohol and drug use composite score from the Addiction Severity Index (ASI) which have demonstrated high concordance with DSM-IV substance use disorders [42], and days abstinent in the previous 30-day period. All substance use data were collected at baseline and at each follow-up wave.

Internal consistency for the drugs composite score in this sample was α = 0.60 at baseline, α = 0.60 at the 3-month follow-up, and α = 0.64 at the 9-month follow-up. Sample internal consistency for the alcohol composite score items was α = 0.89 at baseline, α = 0.92 at the 3 month follow-up, and α = 0.77 at the 9-month follow-up.

General aggression was measured using the 12-item version of the Buss-Perry Aggression Questionnaire (BPAQ, [18]), a commonly used measure of general aggression in both general population and forensic samples with good psychometric properties. Sample items include “Given enough provocation, I may hit another person.” And “I often find myself disagreeing with people.” The items are scored on a five-point Likert scale ranging from 1 (“extremely uncharacteristic of me”) to 5 (“extremely characteristic of me).” Sample internal consistency for the BPAQ was α = 0.82 at baseline, α = 0.81 at 3-month follow-up and α = 0.80 at 9-month follow-up.

Interpersonal aggression was measured using the 14-item version of the Self-Report of Aggression and Social Behavior Measure [36], a measure of interpersonal aggressive acts and dispositions. Sample items include “My friends know that I will think less of them if they do not do what I want them to do.” And “When I am mad at a person, I try to make sure she/he is excluded from group activities (such as going to the movies or to a bar).” Items are rated on a five-point Likert scale from 0 (“Never”) to 4 (“Very often”). The internal consistency for the SRASBM in this sample was α = 0.78 at baseline, α = 0.81 at the 3-month follow-up, and α = 0.82 at the 9-month follow-up.

### Blinding

Research technicians not affiliated with the clinics carried out all assessments at the 3 and 9-month follow-up interviews and were blind to treatment group allocation.

### ILC adherence rating

Two independent raters evaluated a sample of the audiotaped ILC sessions for manual adherence. Adherence was rated on a Likert scale from 1 to 5, in which 1 indicates low adherence (that the session is largely independent of the manual), and 5 indicates high adherence (that the counsellor follows the manual closely).

### Calculation of sample size

Assuming equal numbers of participants in intervention and control arms, a correlation of 0.60 between the same measure collected at two different points in time, and two follow-up waves, a total sample size of 146 was calculated for an effect size of 0.40, an alpha level of 0.05, and a power of 0.80 [19]. To adjust for potential attrition at follow-up, we aimed to include 200 patients in the study.

#### Data analysis

The two groups were compared in terms of baseline characteristics using *χ*^2^ tests for dichotomous variables, and t-tests for continuous variables.

We report means and standard deviations for dependent variables at baseline, the 3 and 9-month follow-ups, and standard mean differences [SMD] between baseline and each follow-up wave within both groups. The SMD was calculated as the difference between baseline and follow-up mean, divided by the baseline standard deviation for the group. It is common to describe an SMD of 0.2 as small, 0.5 as medium, and 0.8 as a large effect size, following Cohen [10]. Fixed-effects regression analysis was used to assess the statistical significance of within-group changes.

Random-effects regression was used to assess the effects on substance use and aggression at the 3 and 9-month follow-up points after randomization. The outcome analyses were by intent-to-treat, i.e. analyzed by randomization arm irrespective of attendance or treatment compliance. Random effects were estimated for both patient and site, and covariates were gender, age, and receiving substitution treatment at baseline. The predictors were randomization status and assessment wave, and the interaction of randomization status and assessment wave. All analyses were controlled for opioid substitution treatment because such medication may substantially influence both illicit drug use and use of treatment services, adding significant variance to the dependent variables in ways that could potentially mask effects of treatment.

Patients were included in the outcome analyses if they had complete data at baseline and at least one follow-up. For days abstinent, we also report the proportion at each follow-up wave who reported no days of substance use (i.e., current abstinence) and the proportion who reported use all days (i.e., 30 days of use in the past 30 days).

## Results

**Fig. 1:**
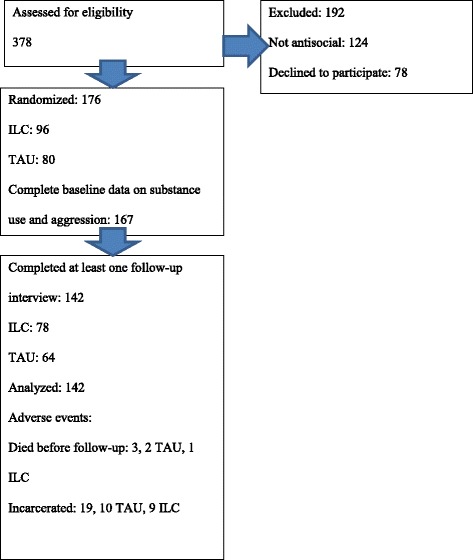
Flow diagram

The flow of patients through the trial is illustrated in Fig. 1.

A total of 142 patients had complete data, including at least one follow-up wave, and could be included in the analyses, of which 64 were assigned to TAU and 78 to ILC. The sample was 87 % male, the mean age was 32.21 years of age ([SD] = 8.90), and 36.5 % received opioid substitution treatment at the time of randomization.

The most commonly used drugs in the past 30 days were cannabis (69.9 %), alcohol (67.1 %), benzodiazepines (41.8 %) and opioids (41.3 %).

### Baseline equivalence

We compared the ILC and TAU groups at baseline, regardless of whether they had been re-interviewed or not. The descriptive statistics are shown in Additional file 1: Table S1.

There were no significant differences in terms of drugs or alcohol composite scores, PDA, or aggression scores at baseline. However, patients in the ILC group reported more days of amphetamine use than TAU patients (*p* < 0.05), and were more often abstinent in the past month at baseline (*χ*^2^(1) = 3.52, *p* = 0.029).

### Exposure to the intervention

Of the 96 patients originally randomized to the ILC group, 71 (76 %) attended at least one session, and 22 (23 %) attended all six sessions. The median number of sessions attended was 2, and the mean was 2.8 (SD = 2.4). For patients who attended the first session, the median ILC time before the first session was 26 days (inter-quartile range: 13 to 59 days). A total of 39 different counsellors delivered the ILC intervention.

### Manual adherence

Of the 80 audiotaped sessions rated, 20.3 % were rated as a 5 on the adherence scale, 72.2 % were rated as a 4, and 7.6 % were rated as a 2 or 3.

### Follow-up

The follow-up rate at 3 months was 79 % (81 % in the TAU group and 77 % in the ILC group) and 69 % at 9 months (71 % in the TAU group and 68 % in the ILC group). Of all follow-up interviews, 75 % were conducted at substance abuse treatment clinics, 9 % in the interviewees’ homes, 3 % in prisons, 12 % in other places, 1 % were conducted as telephone interviews, and 12 % were conducted in various other places, including cafés, and public libraries.

### Attrition analyses

We compared patients with at least one follow-up interview with patients who had never been re-interviewed in terms of age, gender, drugs and alcohol composite score, adult ASPD criteria, substitution treatment at baseline, and in terms of intake unit. No differences between patients interviewed and patients lost to follow-up attained statistical significance, except that there was a difference between clinics in terms of follow-up rate (*χ*^2^(12) = 25.7, *p* = 0.012). Rates of patients interviewed at least once ranged from 63 % in some clinics to 100 % in others.

### Substance use outcomes

Mean values for substance use variables in both groups at all assessment waves are summarized in Table 1. Each row contains the mean and standard deviation at each assessment wave for each group, as well as the standard mean difference as an effect size indicating within-group change. In addition to means and standard deviations for the dependent variables and standardized mean differences, Table 1 shows the percentage of patients with past-month abstinence (i.e., percent reporting zero days of use) and the percentage of daily users (percent reporting 30 days of use in the past 30 days).

**Table 1:** Descriptive statistics of substance use at all assessment waves (means and standard deviations)

	TAU					ILC				
	Baseline (*n* = 74)	3 months (*n* = 61)	SMD	9 months (*n* = 55)	SMD	Baseline (*n* = 93)	3 months (*n* = 72)	SMD	9 months (*n* = 63)	SMD
ASI drugs CS	0.19 (0.13)	0.21 (0.12)	−0.12	0.16 (0.13)	0.27**	0.20 (0.13)	0.17 (0.12)	0.23*	0.15 (0.12)	0.42**
ASI alcohol CS	0.15 (0.22)	0.12 (0.22)	0.14	0.10 (0.18)	0.22*	0.14 (0.22)	0.12 (0.22)	0.22	0.12 (0.21)	0.10
Days Abstinent	11.8 (11.7)	10.8 (11.2)	−0.09	13.7 (12.7)	0.17	9.07 (10.9)	13.2 (12.7)	0.38*	15.3 (13.3)	0.58**
Abstinent	12 %	13 %		13 %		3 %	17 %		21 %	
Daily use	36 %	36 %		33 %		42 %	37 %		31 %	

Abstinent indicates zero days of illicit drug or alcohol use out of the past 30. Significant differences in fixed-effects regression: *: *p* < 0.05. **p < 0.01. Nine participants had not completed data on substance use at baseline

*TAU* Treatment as usual, *ILC* Impulsive lifestyle counselling, *ASI* Addiction Severity Index, *CS* Composite score, *SMD* standard mean difference

The ILC group had reduced their drug use composite score by a small effect size at 3 months (SMD = 0.23, *p* = 0.042) and at 9 months (SMD = 0.42, *p* = 0.000). The TAU group increased their drug use composite score by a small effect size at 3 months (SMD = −0.12, ns) and reduced it by a small effect size at 9 months (SMD = 0.27, *p* = 0.001).

The ILC group reduced their alcohol use composite score by a small effect size (SMD = 0.22, ns) at 3 months and at 9 months (SMD = 0.11, ns). The TAU group reduced their alcohol use severity with a small effect size (SMD = 0.14, ns) at 3 months and at 9 months (SMD = 0.22. *p* = 0.020).

The ILC group increased their days abstinent with a small effect size at 3 months (SMD = 0.38, *p* = 0.042) and a moderate effect size at 9 months (SMD = 0.58, *p* < 0.001). The TAU group reduced their days abstinent by a small effect size at 3 months (-0.09, ns) and increased it by a small effect size by at 9 months (0.17, ns).

The proportion of patients who reported being completely abstinent was stable in the TAU group, at 12 % at baseline, and 13 % at 3 months follow-up and at 9 months follow-up. In the ILC group, 3 % were abstinent at baseline, 17 % at 3 months and 21 % at 9 months. As for daily use, the TAU group reported 36 % at baseline and 36 % and 33 % at the two follow waves. In the ILC group 42 % reported daily use at baseline and 37 and 31 % did so at the follow up waves.

The results of mixed effects regression on substance use outcomes are summarized in Table 2. For each dependent variable, Table 2 reports the effects of assessment wave for 3 and 9 months follow-up, and the interaction between randomization and assessment wave for both waves. Additionally, the table contains the intraclass correlations for randomization site and patient with 95 % confidence intervals, and the model Wald *χ*^2^. For the ASI drugs composite score, the Wald *χ*^2^ was 93.90 (df = 8, *p* < 0.001). Patients randomized to the ILC group had significantly less drug use at the 3-month follow-up compared to the TAU group (β =−0.041, p = 0.018). In addition to the ILC and time variables, patients who received opioid substitution treatment at baseline had more severe drug use (β = 0.11, *p* < 0.001).

**Table 2:** Results of mixed effects regression on substance use outcomes (*n* = 142)

Dependent variable		Coefficient	95 % CI	P-value
ASI Drugs CS	ILC intercept	0.001	−0.026 to 0.046	0.588
	3 months	0.015	−0.017 to 0.047	0.362
	9 months	−0.052	−0.086 to−0.019	0.002
	ILC X 3 months	−0.052	−0.096 to−0.009	0.018
	ILC X 9 months	−0.004	−0.049 to 0.042	0.872
	ICC site	0.017	0.000 to 0.361	
	ICC Patient	0.335	0.234 to 0.455	
	Wald *χ* ^2^(8)	93.90		0.000
ASI Alcohol CS	ILC intercept	−0.012	−0.077 to 0.052	0.708
	3 months	−0.041	−0.092 to 0.010	0.134
	9 months	−0.066	−0.120 to−0.112	0.016
	ILC X 3 months	0.008	−0.061 to 0.077	0.814
	ILC X 9 months	0.049	−0.023 to 0.121	0.182
	ICC Site	0.072	0.016 to 0.272	
	ICC Patient	0.506	0.402 to 0.610	
	Wald *χ* ^2^(8)	16.24		0.039
Days abstinent	ILC intercept	−2.300	−6.179 to 1.580	0.245
	3 months	0.970	−4.027 to 2.088	0.534
	9 months	2.359	−0.863 to 5.810	0.151
	ILC X 3 months	4.319	0.183 to 8.456	0.041
	ILC X 9 months	3.584	−0.751 to 7.919	0.105
	ICC Site	0.005	0.000 to 1.000	
	ICC Patient	0.471	0.369 to 0.576	
	Wald *χ* ^2^(8)	31.17		0.000

All analyses adjusted for site and individual, gender, age, and substitution at baseline

*TAU* Treatment as usual, *ILC* Impulsive lifestyle counselling, *ASI* Addiction Severity Index, *CS* Composite score, *ICC* Intraclass correlation

For the alcohol composite score, the Wald *χ*^2^ was 16.24 (df = 8, *p* = 0.039), and there was no significant effect of randomization at either follow-up wave. The whole group decreased their level of alcohol problems at 9 months (*p* = 0.011).

For days abstinent, the Wald *χ*^2^ was 31.17, (df = 8, *p* < 0.001), and patients randomized to the ILC group reported 4.3 more days abstinent at 3 month follow-up (CI: 0.18 to 8.46). Additionally, patients who received substitution treatment had fewer days abstinent (β = −5.64, *p* = 0.003).

### Aggression outcomes

Mean values for aggression variables in both groups at all assessment waves are summarized in Table 3. Table 3 is presented similarly to Table 1.

**Table 3:** Descriptive statistics of aggression at all assessment waves for patients (means and standard deviations)

	TAU					ILC				
	Baseline (*n* = 74)	3 months (*n* = 61)	SMD	9 months (*n* = 55)	SMD	Baseline (*n* = 93)	3 months (*n* = 70)	SMD	9 months (*n* = 63)	SMD
BPAQ	4.44 (1.20)	3.83 (1.16)	0.50**	3.52 (1.25)	0.76**	4.38 (1.10)	4.01 (1.16)	0.34**	3.59 (1.05)	0.72**
SRASBM	1.00 (0.63)	0.64 (0.46)	0.57**	0.61 (0.52)	0.61**	0.93 (0.61)	0.64 (0.49)	0.47**	0.47 (0.39)	0.75**

Nine participants had not completed data on aggression at baseline

*TAU* Treatment as usual, *ILC* Impulsive lifestyle counselling, *BPAQ* Buss-Perry Aggression Questionnaire, *SRASBM* Self-Report of Aggression and Social Behavior Measure, *SMD* standard mean difference ** p < 0.01

The ILC group reduced their general aggression (BPAQ) with a moderate effect size at 3 months (SMD = 0.51, *p* < 0.001) and a large effect size at 9 months (SMD = 0.76, *p* < 0.001). The TAU group reduced their general aggression with a moderate effect size at 3 months (SMD = 0.50, *p* < 0.001) and increased it by a large effect size by at 9 months (0.76, *p* < 0.001).

The ILC group reduced their interpersonal aggression (SRASBM) with a small effect size at 3 months (SMD = 0.57, *p* < 0.001), and at 9 months (SMD = 0.72, *p* < 0.01). The TAU group reduced their interpersonal aggression with a moderate effect size at 3 months (SMD = 0.57, *p* < 0.001) and a moderate effect size at 9 months (SMD = 0.61, *p* < 0.001).

The results of mixed effects regression on outcomes are summarized in Table 4. Similar to Table 2, for each dependent variable, Table 4 reports the effects of assessment wave for 3 and 9 months follow-up, and the interaction between randomization and assessment wave for both waves, the intraclass correlations for randomization site and patient with 95 % confidence intervals, and the model Wald *χ*^2^. For the BPAQ, the Wald *χ*^2^ was 101.82 (df = 8, *p* < 0.001). No differences were found between ILC and TAU at any point, but across both groups, considerable reductions in general aggression were observed at both follow-up waves.

**Table 4:** Results of mixed effects regression on aggression outcomes (*n* = 142)

Dependent variable		Coefficient	95 % CI	*P*-value
Buss-Perry Aggression Questionnaire	ILC intercept	−0.121	−0.500 to 0.258	0.533
	3 months	−0.693	−0.946 to−0.440	0.000
	9 months	−0.967	−1.234 to−0.700	0.000
	ILC X 3 months	0.334	−0.001 to 0.677	0.056
	ILC X 9 months	0.199	−0.161 to 0.558	0.279
	ICC site	0.000	0.000 to 0.000	
	ICC Patient	0.620	0.532 to 0.702	
	Wald *χ* ^2^(8)	99.24		0.000
SRASBM	ILC intercept	−0.084	−0.256 to 0.088	0.339
	3 months	−0.392	−0.522 to−0.261	0.000
	9 months	−0.460	−0.597 to−0.322	0.000
	ILC X 3 months	0.083	−0.092 to 0.260	0.351
	ILC X 9 months	0.026	−0.158 to 0.210	0.782
	ICC Site	0.000	0.000 to 0.000	
	ICC Patient	0.513	0.413 to 0.612	
	Wald *χ* ^2^(8)	116.95		0.000

For the SRASBM, the Wald *χ*^2^ was 124.43 (df = 8, *p* < 0.001), and there was no significant effect of randomization at either follow-up wave. No differences were found between ILC and TAU at any point, but across both groups considerable reductions in interpersonal aggression were observed at both follow-up waves.

## Discussion

This trial provides the first evidence that the ILC program, a short-term, highly structured psychoeducational intervention, increases the efficacy of treatment for substance use disorders for patients with comorbid ASPD and substance use disorder.

At the 3-month follow-up, patients who had been randomized to the ILC group had increased days abstinent compared to patients randomized to TAU and had less severe drug use. As is typical in community substance abuse treatment contexts, attendance to the intervention was less than perfect. In spite of this, the intention-to-treat analysis supported the benefits of the treatment.

No statistically significant effects were observed for self-reported aggression. Across both groups, substantial decreases in self-reported aggression were observed at both follow-up waves, indicating either that participation in substance abuse treatment reduced aggression, or alternatively a regression to the mean effect [7], or a retest artefact [45]. It is plausible that the intervention was too brief to have an impact on aggressive behavior. However, the findings are similar to those of a somewhat more intensive intervention with cognitive behavioral therapy for psychiatric outpatients with ASPD, where some effect on substance use was observed, but none on self-reported aggression [16].

The brief non-intensive intervention delivered in this study is not a cure for ASPD, but can constitute one of many small steps towards improving treatment for this under-served population. Substance use is a factor that complicates the treatment of any psychiatric disorder, and if substance use is reduced, it opens up the possibility of further interventions and support, potentially increasing the patient’s social and psychological stability.

As in several previous studies, we found evidence that individuals with ASPD reduced substance use during standard substance abuse treatment [1, 39]; in the entire sample, alcohol and drugs composite scores were significantly reduced at the 9-month follow-up. These findings add further support to the view that individuals with ASPD should not be excluded from substance abuse treatment [28, 34, 39]. On the other hand, reductions in substance use generally represented small effect sizes.

Considering the results in Table 1, differences between the two groups were more pronounced in terms of patients remaining completely abstinent, than in terms of patients using non-prescription drugs or alcohol daily; in both the ILC and the TAU condition, the proportion of patients using substances daily was stable over time, whereas the proportion of patients that were abstinent increased in the ILC group.

The ILC program did not have a significant impact on the alcohol composite score. In fact, the control group had reduced their level of alcohol severity by a small effect size at 9 months, whereas the ILC group was virtually unchanged. However, given that it did not attain statistical significance, speculating about the reasons for this negative finding is unwarranted.

The current trial supported the findings by Davidson and colleagues that substance use could be influenced by targeting ASPD using a psychosocial intervention [16].

An additional finding from this study was that the group of patients who received opioid substitution medication at baseline had more severe drug problems and fewer days abstinent over the course of the trial. This finding does not necessarily indicate that substitution treatment is ineffective, as it may just as well reflect pre-existing higher severity of drug problems in the opioid substitution group.

### Limitations and strengths

Several limitations for this study must be acknowledged. First, we had only self-reported data on substance use with no biological verification available. It is no longer standard to use biological data for collecting data on drug use (e.g. [17, 30]), and a number of studies have indicated that biological verification underestimates substance use and does not provide more valid data than self-reporting (e.g. [12, 51, 52]). Another limitation is that the patient population in this study was heterogeneous, including patients using a wide range of substances, and with a wide age range. On the other hand, this means that most patients in substance abuse treatment with ASPD would meet inclusion criteria and could thus/potentially increase the generalizability of the findings [29, 57].

A further limitation is that we were not able to implement a standard of treatment as usual. The participating clinics represented a wide range of rural and urban settings, and the service level depended on the local authorities and the division of labor between local psychiatric, social, and substance abuse treatment services, making it infeasible to standardize the treatment. Since the intention of ILC is to increase help-seeking behavior and compliance with treatment, it did not seem pertinent to control for overall amount of services received.

Further, the absence of an attention placebo condition meant that the efficacy of the study may be overestimated in the present analyses.

The principle strength of this study lies mainly in its pragmatic approach to implementing screening and intervention in a way that is practically feasible at busy clinics [17]. The intervention was specifically selected to require minimal training of clinicians, yet had a notable impact on the reduction of substance use.

Although this study adds to the development of evidence-based targeted interventions for personality disorders and ASPD, further research is needed to assess the relative usefulness and credibility of psychoeducative programs such as Impulsive Lifestyle Counselling or the models used by Banerjee et al. [6] or Zanarini and Frankenburg [56] for patients with ASPD.

Of particular interest would be studies with a focus on ASPD that examine different approaches at substance abuse treatment services and compare them to other services offered to patients with ASPD in terms of their efficacy, their costs and the ease of implementation.

## Conclusion

Impulsive lifestyle counselling, a brief psychoeducative intervention, had an impact on substance use in terms of drug use problems and days abstinent for outpatients with substance use disorders and antisocial personality disorder. More research is needed on how to provide optimal treatment for people with antisocial personality disorder.

## Additional file

Additional file 1: Table S1. Descriptive statistics at baseline (means and standard deviations). (DOCX 14 kb)
