# Supplementary material for: Psycho-education for substance use and antisocial personality disorder: a randomized trial
Source: BMC Psychiatry. 2015 Nov 14;15:283. doi: 10.1186/s12888-015-0661-0 (PMC4647713; doi:10.1186/s12888-015-0661-0)
Supplement: Additional file 1: Table S1. — Descriptive statistics at baseline (means and standard deviations). (DOCX 14 kb) [file 12888_2015_661_MOESM1_ESM.docx]

Table S1. Descriptive statistics at baseline (means and standard deviations)

|  | TAU | ILC | P-value |
| --- | --- | --- | --- |
| Days of substance use |  |  |  |
| Alcohol use | 6.46 (8.45) | 4.81 (6.97) | 0.225 |
| Alcohol, +5 units per day | 3.44 (5.92) | 3.16 (5.61) | 0.784 |
| Heroin | 1.48 (5.15) | 0.49 (2.75) | 0.164 |
| Methadone | 7.20 (12.83) | 7.73 (12.87) | 0.817 |
| Other opioids | 0.92 (4.27) | 0.83 (3.26) | 0.901 |
| Tranquilizers | 8.00 (12.26) | 7.42 (11.93) | 0.786 |
| Cocaine | 1.31 (3.91) | 1.31 (4.38) | 0.995 |
| Amphetamines | 0.36 (0.83) | 1.43 (3.83) | 0.037 |
| Cannabis | 13.54 (12.86) | 24.26 (13.09) | 0.755 |
| Hallucinogenes | 0.14 (0.71) | 0.04 (0.26) | 0.284 |
| Inhalants | 0.00 (0.00) | 0.01 (0.12) | 0.371 |
| Buprenorphine | 2.78 (8.44) | 1.64 (6.70) | 0.390 |
| Poly-substance | 6.77 (10.52) | 7.57 (11.58) | 0.687 |
| Conduct disorder criteria | 3.90 (0.80) | 4.04 (0.83) | 0.337 |
| Adult antisocial behaviors | 4.87 (1.07) | 5.14 (0.98) | 0.120 |

Notes:

TAU: Treatment as usual. ILC: Impulsive lifestyle counselling. ASRS: ADHD-Self-report scale. Conduct disorder criteria and adult antisocial behaviors are both counted based on the antisocial personality disorder module of the Mini International Neuropsychiatric Interview.
